# Supplementary material for: How do international humanitarian aid workers stay healthy in the face of adversity?
Source: PLoS One. 2022 Nov 16;17(11):e0276727. doi: 10.1371/journal.pone.0276727 (PMC9668143; doi:10.1371/journal.pone.0276727)
Supplement: S1 Appendix — (DOCX) [file pone.0276727.s001.docx]

**Appendix A**

Correlations between health outcome variables and predictor variables.

*Anxiety*

*Note. * p* = <.05; *** p* = <.01.

*Depression*


*Note. * p* = <.05; *** p* = <.01.

*Emotional exhaustion* *Note. * p* = <.05; *** p* = <.01.

*PTSD*


*Note. * p* = <.05; *** p* = <.01.

*Work engagement*


*Note. * p* = <.05; *** p* = <.01.
