# Supplementary material for: How do international humanitarian aid workers stay healthy in the face of adversity?
Source: PLoS One. 2022 Nov 16;17(11):e0276727. doi: 10.1371/journal.pone.0276727 (PMC9668143; doi:10.1371/journal.pone.0276727)
Supplement: S2 Appendix — (DOCX) [file pone.0276727.s002.docx]

**Appendix B**

Humanitarian Field Stressor List

| Reported stress level | Significant | | High | |
| --- | --- | --- | --- | --- |
|  | *n* | *%* | *n* | *%* |
| *Conditions in the field* |  |  |  |  |
| Location/travel | 77 | 16.0 | 22 | 4.6 |
| Climate | 80 | 16.6 | 38 | 7.9 |
| Security context of the country | 74 | 15.4 | 36 | 7.5 |
| Separation from family and friends | 59 | 12.2 | 23 | 4.8 |
| Housing/sanitation | 40 | 8.3 | 19 | 3.9 |
| Food/amenities | 39 | 8.1 | 13 | 2.7 |
| Health risks | 27 | 5.6 | 12 | 2.5 |
| *Cultural stressors* |  |  |  |  |
| Language problems with beneficiaries | 50 | 10.4 | 11 | 2.3 |
| Local customs or mentality | 53 | 11.0 | 14 | 2.9 |
| Cultural sensitivity of MSF colleagues | 46 | 9.6 | 15 | 3.1 |
| *Work stressors* |  |  |  |  |
| Unclear communication in the project | 79 | 16.4 | 48 | 10.0 |
| High workload | 78 | 16.2 | 44 | 9.1 |
| Not available staff/replacements | 71 | 14.8 | 37 | 7.7 |
| Emotional impact of the work | 64 | 13.3 | 28 | 5.8 |
| Role of the project coordinator (PC)/medical team leader (MTL) | 58 | 12.1 | 43 | 8.9 |
| Security and safety conditions | 51 | 10.6 | 21 | 4.4 |
| Lack of management appreciation | 53 | 11.0 | 39 | 8.1 |
| Lack of technical support | 54 | 11.2 | 26 | 5.4 |
| Role of the country management team (CMT) | 51 | 10.6 | 30 | 6.2 |
| Role of HQ/Operations | 40 | 8.3 | 24 | 5.0 |
| Unclear/non-existent job profile or assignment | 33 | 6.9 | 26 | 5.4 |
| Powerlessness/hopelessness | 34 | 7.1 | 15 | 3.1 |
| Poor working conditions | 29 | 6.0 | 8 | 1.7 |
| Contact with authorities | 28 | 5.8 | 4 | .8 |
| Working with national staff | 17 | 3.5 | 8 | 1.7 |
| *Team stressors* |  |  |  |  |
| Problematic communication in the team | 67 | 13.9 | 38 | 7.9 |
| Negative team atmosphere | 50 | 10.4 | 30 | 6.2 |
| Conflicts in the team | 44 | 9.1 | 25 | 5.2 |
| Poor/no relationships with other team members | 43 | 8.9 | 20 | 4.2 |
| Breaching of code of conduct by others | 31 | 6.4 | 11 | 2.3 |
| Team composition (e.g., gender, cultures, composition) | 23 | 4.8 | 13 | 2.7 |
| *Self-experienced upsetting events* |  |  |  |  |
| Security and safety incidents | 42 | 8.7 | 9 | 1.9 |
| Looting/hold-up/assault/shooting | 23 | 4.8 | 15 | 3.1 |
| Intimidation by authorities | 18 | 3.7 | 11 | 2.3 |
| Hearing or seeing violence/intimidation/abuse | 37 | 7.7 | 20 | 4.2 |
| *Code of conduct* |  |  |  |  |
| Experienced intimidation/aggression by colleagues | 28 | 5.8 | 13 | 2.7 |
| Witnessed intimidation/aggression by colleagues | 22 | 4.6 | 12 | 2.5 |
| Witnessed sexual harassment/violence toward colleagues | 8 | 1.7 | 10 | 2.1 |

*Note.* Reported levels of experienced stress per item in the two highest response categories. Table reported in (1).
