# Supplementary figures and images for: How do international humanitarian aid workers stay healthy in the face of adversity?
Source: PLoS One. 2022 Nov 16;17(11):e0276727. doi: 10.1371/journal.pone.0276727 (PMC9668143; doi:10.1371/journal.pone.0276727)

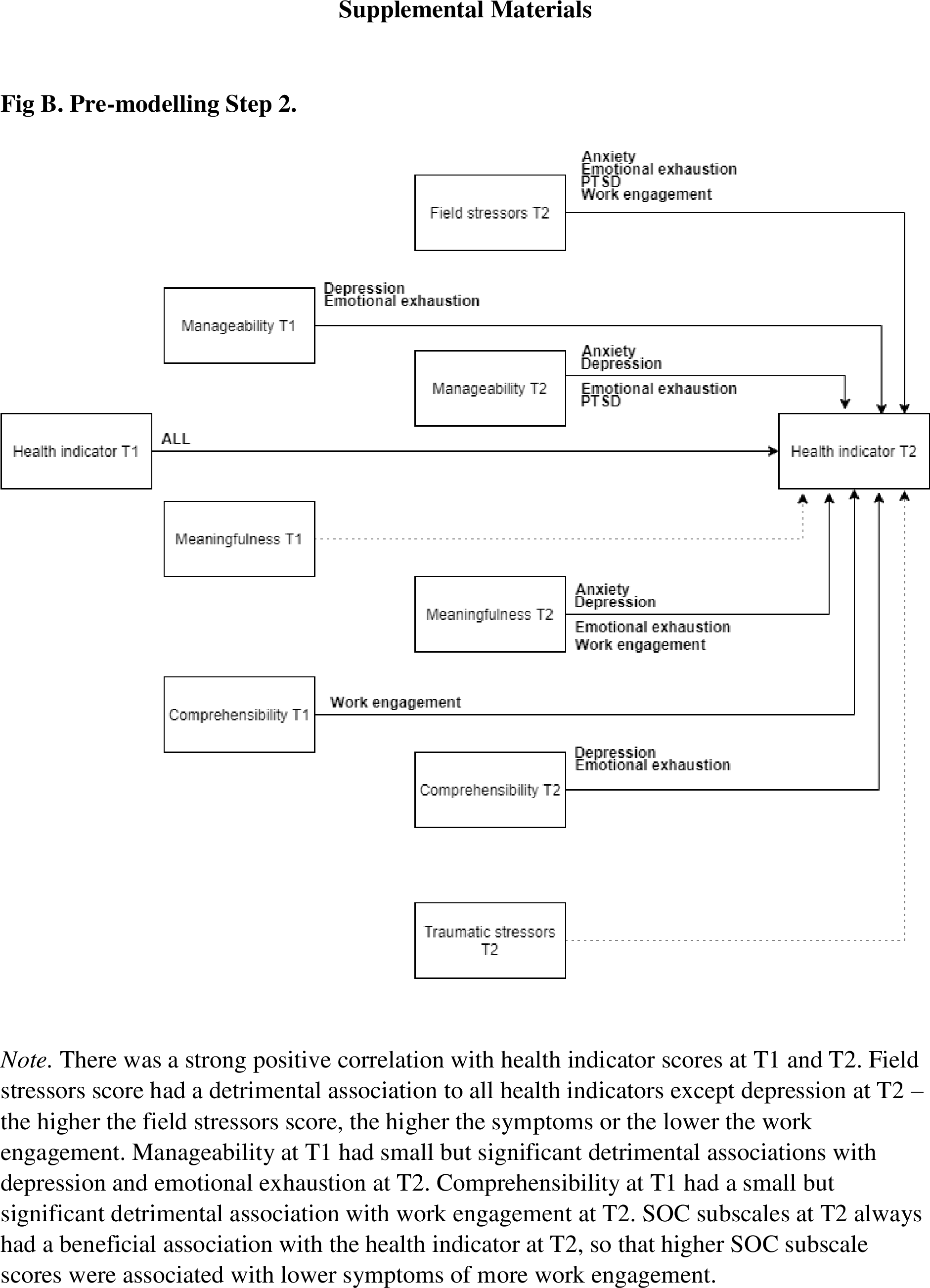

Supplement: S1 File — A. Pre-modelling Step 1. B. Pre-modelling Step 2. C. Pre-modelling Step 3. (ZIP) [file pone.0276727.s003.zip › Supplementary Fig B. Step 2.tif]

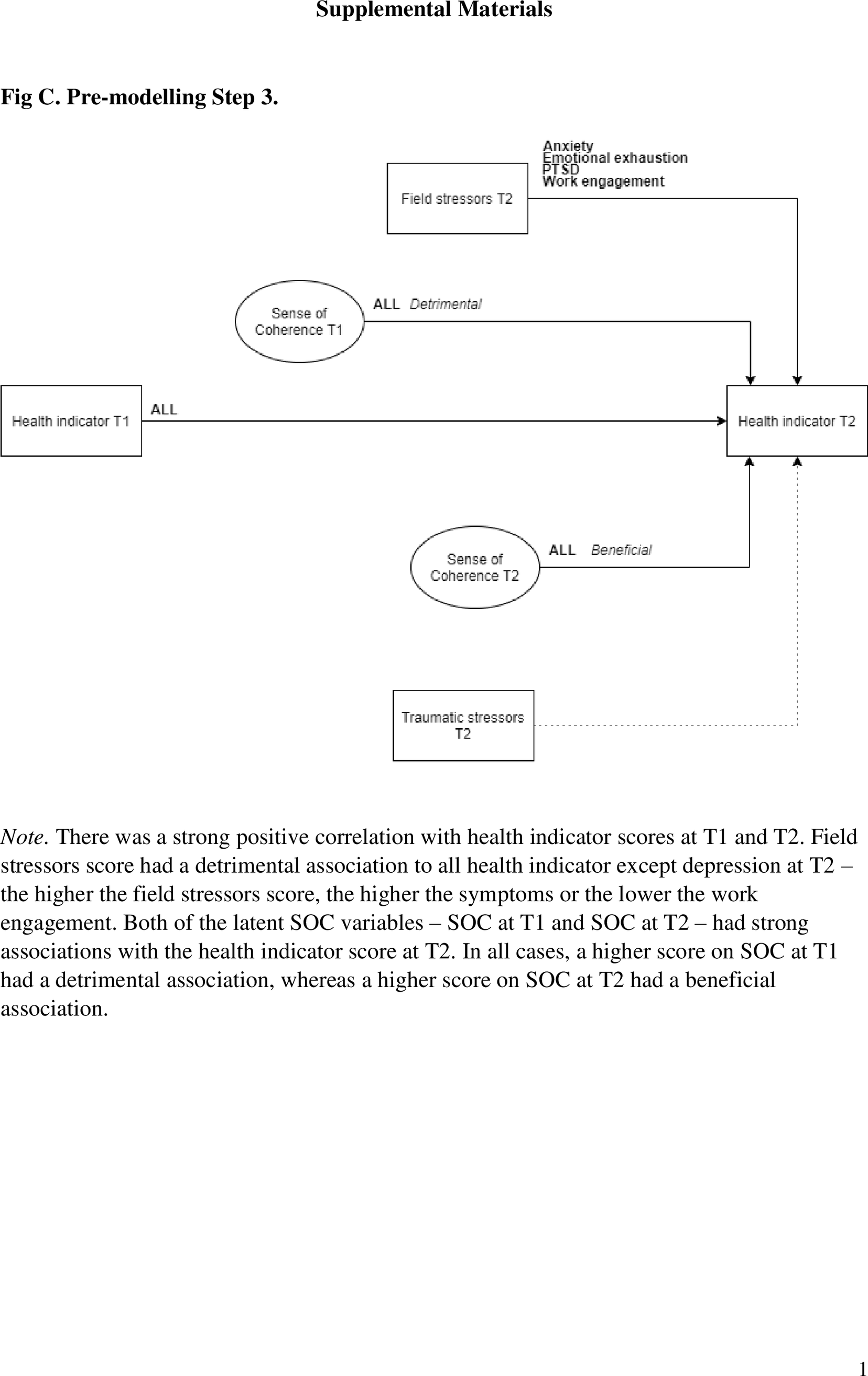

Supplement: S1 File — A. Pre-modelling Step 1. B. Pre-modelling Step 2. C. Pre-modelling Step 3. (ZIP) [file pone.0276727.s003.zip › Supplementary Fig C. Step 3.tif]

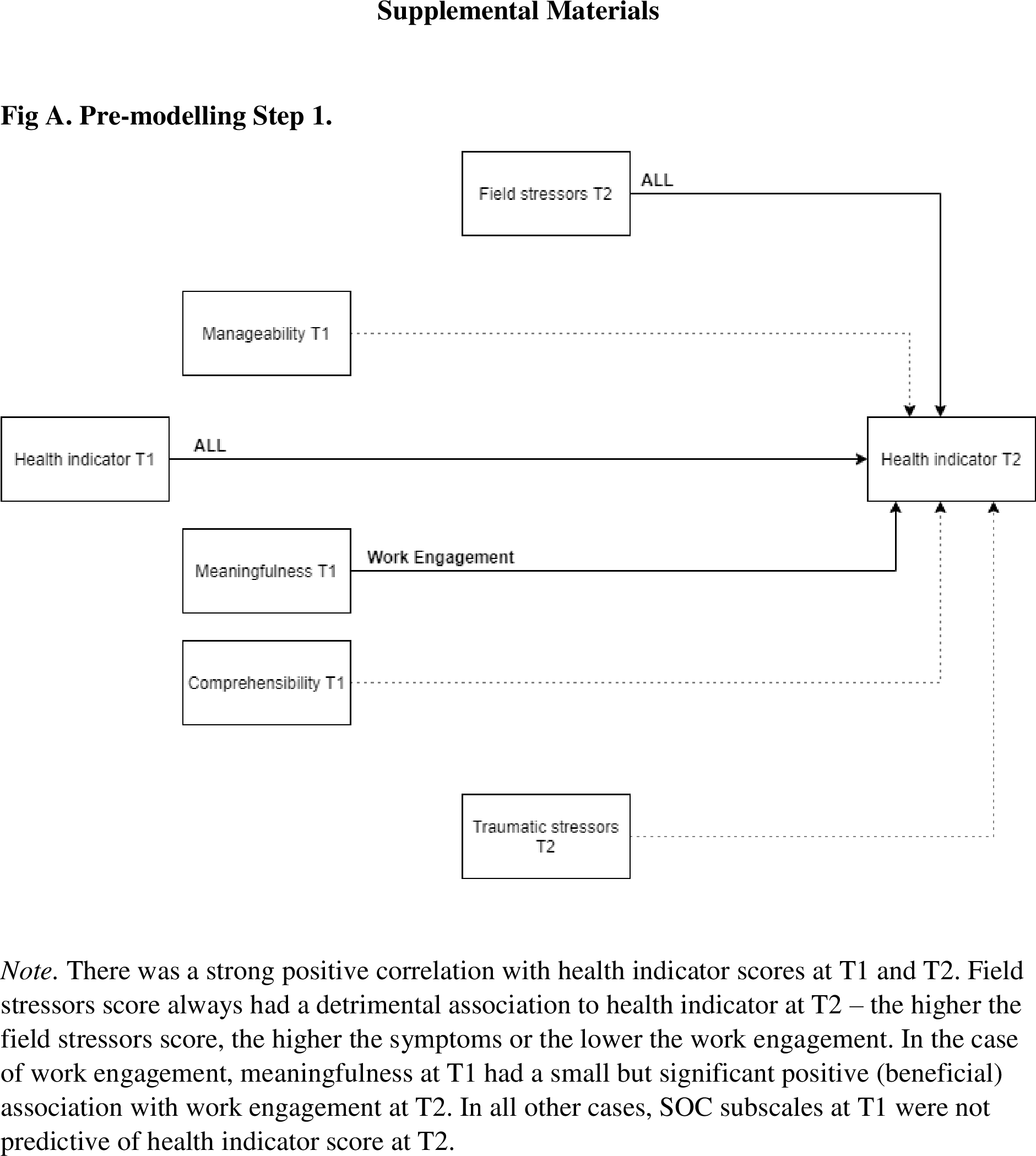

Supplement: S1 File — A. Pre-modelling Step 1. B. Pre-modelling Step 2. C. Pre-modelling Step 3. (ZIP) [file pone.0276727.s003.zip › Supplementery Fig A. Step 1.tif]
