# Supplementary material for: How do international humanitarian aid workers stay healthy in the face of adversity?
Source: PLoS One. 2022 Nov 16;17(11):e0276727. doi: 10.1371/journal.pone.0276727 (PMC9668143; doi:10.1371/journal.pone.0276727)
Supplement: Attachment — Submitted filename: PLOS ONE Rebuttal FINAL 1 Aug 2022.docx [file pone.0276727.s004.docx]

**PONE-D-21-37102**

**How do international humanitarian aid workers stay healthy in the face of adversity? Is Sense of Coherence a mediator for health and work engagement?**

Amsterdam, 8 August 2022

**EDITOR GENERAL COMMENTS**

When submitting your revision, we need you to address these additional requirements.

**1. Please ensure that your manuscript meets PLOS ONE's style requirements, including those for file naming. The PLOS ONE style templates can be found at**

**Reply**

We made changes in the document to meet PLOS ONE’s style requirements.

**2. We note that the grant information you provided in the ‘Funding Information’ and ‘Financial Disclosure’ sections do not match.**

**When you resubmit, please ensure that you provide the correct grant numbers for the awards you received for your study in the ‘Funding Information’ section.**

**Reply**

MSF Operational Centre Amsterdam funded the study. It is part of the regular research budget and as such not allocated with a specific grant number.

**3. Thank you for stating the following in the Competing Interests/Financial Disclosure* (delete as necessary) section.**

**Reply**

We added to the section on Declaration of conflicting interests the Editor’s suggested text and added a further detailed description of the way the independence was managed.

**We added to the section on ‘Declaration of Interest’ the following. This text we also included in the rebuttal letter to the Editor.**

*Dr. Kaz de Jong is employed by Medecins Sans Frontiers (MSF) the research funder. To ensure no potential conflicts could arise, the data collection and statistical analyses were performed by independent researchers without involvement of dr. de Jong or any other employee of MSF. The results were interpreted with the full research team, including independent researchers and external supervisors (university professors, RK, IK) to ensure quality and independence. MSF also signed a written agreement that it would adhere to the code of conduct for scientific integrity of the Royal Netherlands Academy of Arts and Sciences regarding this research. Both the funding of MSF as well as a clear description of the role of the financing body is mentioned in a separate statement in the manuscript. The role of the different authors in the research is described in detail in the manuscript.*

**4. We note that one or more of the authors are employed by a commercial company: name of commercial company.**

**Reply**

There is a misunderstanding concerning the employment of the authors for a commercial company. All authors are employed by Not-For-Profit organisations: Medecins sans Frontiers, HealthNet TPO, ARQ Centre of Expertise for the Impact of Disasters and Crises, and the Utrecht University. None of these organizations have a commercial interest.

Please take note of the different websites of the different organisations:

*ARQ Impact*

<https://www.impact-kenniscentrum.nl/en>

### Please check out the following Public Benefit Organisation (PBO) statement (also known as a ANBI statement in Dutch): <https://www.arq.org/sites/default/files/domain-9/documents/stichting_arq_anbi_2019-01-9-15465278231035806768.pdf>

Translated: ARQ Centrum ‘45 is registered at the tax paying office as Public Benefit Organisation (ANBI).The ANBI status is described as: A Public Benefit Organisation (PBO) is a foreign ANBI, which means in Dutch: [Algemeen Nut Beogende instelling (ANBI)](https://www.belastingdienst.nl/wps/wcm/connect/bldcontentnl/belastingdienst/zakelijk/bijzondere_regelingen/goede_doelen/algemeen_nut_beogende_instellingen/belastingregels_algemeen_nut_beogende_instellingen). An organisation can only be an ANBI if it’s organisation’s efforts are almost entirely committed to the public benefit.

*HealthNet TPO*

<https://www.healthnettpo.org/en/donate>

# Please check the end of the webpage which states both ANBI status (see above explanation) and CBF status. The Netherlands Fundraising Regulator (CBF) is an independent foundation which has been monitoring fundraising by charities since 1925. The CBF’s task is to promote trustworthy fundraising and expenditure by reviewing fundraising organizations and giving information and advice to government institutions and the public. When a charity has been recognized as a charity by the CBF (CBF-Erkend Goed Doel), you can trust that the organization has been closely reviewed.

**A.**  **Please provide an amended Funding Statement declaring this commercial affiliation, as well as a statement regarding the Role of Funders in your study. If the funding organization did not play a role in the study design, data collection and analysis, decision to publish, or preparation of the manuscript and only provided financial support in the form of authors' salaries and/or research materials, please review your statements relating to the author contributions, and ensure you have specifically and accurately indicated the role(s) that these authors had in your study. You can update author roles in the Author Contributions section of the online submission form.**

**Please also include the following statement within your amended Funding Statement.**

**“****The funder provided support in the form of salaries for authors [insert relevant initials], but did not have any additional role in the study design, data collection and analysis, decision to publish, or preparation of the manuscript. The specific roles of these authors are articulated in the ‘author contributions’ section.”**

**If your commercial affiliation did play a role in your study, please state and explain this role within your updated Funding Statement.**

**Reply**

The funding organisation (Medecins Sans Frontieres) did not play any role in the study design, data collection and analysis, decision to publish, or preparation of the manuscript and only provided financial support in the form of authors' salaries and/or research materials.

**And we changed the Funding Statement section:**

The Management Team of MSF Operational Centre Amsterdam funded the study into the health of its international staff. It was not involved in: study design; collection, statistical analyses, and interpretation of data; writing of the articles; and decisions to submit papers for publication. ARQ Research and Policy Institute Impact independently carried out the collection of the data. The interpretation, (anonymous) analyses, and writing of the article were done by all members of the research team. The MSF principal investigator was not involved in the recruitment, retention, follow-up procedures of the study participants, unaware of the individuals who participated in the study and had only access to anonymized study data. Two scientific experts (Professors) from Utrecht University supervised quality and independence of the scientific process.

**Into the Editor suggested statement**

*The funder provided financial support in the form of a grant that also included salaries for authors (KdJ, SM, HtB, RJK,JFGH, IK). The funder did not have any additional role in the study design, data collection and analysis, decision to publish, or preparation of the manuscript. The specific roles of these authors are articulated in the ‘author contributions’ section.*

**We included in the manuscript a section describing Author Contribution:**

*KdJ         Conceptualization, methodology, interpretation of results, writing original draft, project*

*coordination, funding acquisition*

*SM         Data curation, data collection, methodology, formal analysis, interpretation of results,*

*software, visualisation, writing (review, editing)*

*HtB       Conceptualization, methodology, research quality supervision, writing (review, editing)*

*RK         Conceptualization, methodology, theoretical and research quality supervision, writing*

*(review, editing)*

*JH        Data curation, data collection, methodology, formal analysis, interpretation of results,*

*software management, writing (review, editing)*

*IK        Conceptualization, methodology, formal analysis, research quality supervision, writing*

*(review, editing)*

*All authors approved the final version of the manuscript.*

**b. Please also provide an updated Competing Interests Statement declaring this commercial affiliation along with any other relevant declarations relating to employment, consultancy, patents, products in development, or marketed products, etc.**

**Within your Competing Interests Statement, please confirm that this commercial affiliation does not alter your adherence to all PLOS ONE policies on sharing data and materials by including the following statement: "This does not alter our adherence to  PLOS ONE policies on sharing data and materials.” (as detailed online in our guide for authors** [**http://journals.plos.org/plosone/s/competing-interests**](http://journals.plos.org/plosone/s/competing-interests)**) . If this adherence statement is not accurate and  there are restrictions on sharing of data and/or materials, please state these. Please note that we cannot proceed with consideration of your article until this information has been declared. Please include both an updated Funding Statement and Competing Interests Statement in your cover letter. We will change the online submission form on your behalf.**

**Reply**

None of the authors had any commercial affiliations. They are employed by non-commercial organisations. Therefore, we do not mention commercial affiliations in the rebuttal letter. We hope to have explained sufficiently that none of the authors had commercial affiliations. We kindly ask to proceed with the consideration of this article without information on commercial interests because there are none.

We will mention the funding statement in the rebuttal letter:

*The authors disclosed the following financial support for the research and/or publication of this article: This research was supported by MSF Operational Centre Amsterdam.*

**5. Please ensure that you refer to Figure 3, 5, 7, 9, 11-21 in your text as, if accepted, production will need this reference to link the reader to the figure.**

**Reply**

We have referred to all Tables and Figures in the text and placed the Tables and Figures in the text in accordance with the author guidelines.

**6. Please review your reference list to ensure that it is complete and correct. If you have cited papers that have been retracted, please include the rationale for doing so in the manuscript text, or remove these references and replace them with relevant current references. Any changes to the reference list should be mentioned in the rebuttal letter that accompanies your revised manuscript. If you need to cite a retracted article, indicate the article’s retracted status in the References list and also include a citation and full reference for the retraction notice.**

**Reply**

We have reviewed our reference list to ensure it is complete and correct.

**Following changes were made in the section References:**

I’ve checked through all. All ref. numbers are from the current version (different before editing).

Reference 1: reference updated to reflect where it was published.

Reference 6: Lindstrom added as author.

Reference 9: Authors checked, ‘editor’ removed as no editor for the article.

Reference 21: Salanova added as author.

Reference 42: page numbers corrected.

Reference 48: (N Y) removed from after the journal name

**COMMENTS FROM THE REVIEWERS**

**REVIEWER 1**

**Thank you for the opportunity to review this paper which is about International Humanitarian Aid Workers (iHAWs) and the fact that despite working under stressful and traumatic environments, they remain healthy. The authors used a stress-health model to test whether the concept of a Sense of Coherence (SOC) as a health mechanism would mediate the relationship between stressors related to the field work of iHAWs after they returned from their assignment.

I found this paper very interesting and very well written throughout. The background to the concept and components of SOC was clearly defined as was their rationale for choosing it to explore how and why iHAWs remain relatively healthy despite challenging work and conditions. Salutogenesis was sufficiently explained as the rationale for looking at the three components of SOC.

Methods were clear and I feel their choice of instruments was appropriate to provide the various indicators of health in their model.

Analyses seems comprehensive and appropriate. The number of participants is adequate for the analyses they performed. Although they noted some limitations to further analyses due to sample size. Although not an expert, overall, their statistical analysis performed, and their mediation analysis seems correct. They thoroughly tested the demographic variables, mainly sex, as they may impact on their findings.**

**The discussion again was clearly written and described the results while highlighting how certain variables were mediated by the three components of SOC. I found this very interesting and the I think they have strengthened the importance of considering the context in which any action and behaviour is observed. I feel this information could go beyond the promotion of better health for workers in difficult and challenging environments but can be applied more broadly to situations where the context is specifically unique and/or intense.**

**Reply**

We highly appreciate the comments of the reviewer. We also agree with the comment that our findings can be applied in other unique and/or intense situations. We hope that the publication of this manuscript will enable a larger group to take notice of our findings and consider it for their workplace. We added the following textual change to highlight its potential importance of other populations exposed to similar environments.

**Textual Change in the header Strengths and Limitations (p..).**

*The current health promoting findings also provide avenues of interest for other populations that operate in in similar settings, such as military personnel on deployment.*

**REVIEWER 2**

**Dear authors, my only remark is regarding the last sentence on page 21. In it, you assume that the SOC may not be a stable construct over time, according to your results. I would like to emphasize that the SOC is a heterogeneous construct and this could lead to erroneous conclusions. Please keep in mind that this construct is identical to Generalized Resistance Resources (GRRs), which in practice remain undiscovered by the researchers who measure only the underlying trend for coherence, or SOC. Therefore, measuring pre- and post-assignment SOC in your study is not an indicator of construct instability. During this period, the participants in your study has experienced immeasurable dynamics of GRRs. In my opinion, this explains the discrepancy between pre- and post-assignment SOC in promoting health. I remind you that GGRs includes coping strategies, emotional closeness, knowledge and intelligence, and support system. Refer to: Mittelmark, M. B., Sagy, S., Eriksson, M., Bauer, G. F., Pelikan, J. M., Lindström, B., & Arild Espnes, G. (2017). The handbook of salutogenesis. Springer Nature.**
**In short, I urge you to rethink this sentence in your work. On page 24 of the manuscript, which describes the strengths and limitations, you yourself self-critically point out that the design does not allow for causal conclusions. That is enough !!!**
**I sincerely congratulate you on the idea of researching SOC in these samples, which are so presumptively focused on pathology!**

**Reply**

We appreciate both the comments and congratulations of the reviewer. We partially agree to the comments of the reviewer. Antonovsky (1987) stated SOC, and its underlying GRR remains stable after a certain age (around 30 years). We agree with the reviewer that SOC (the result of the composing GRR, their interactions and balance) may change after exposure to a dramatic different context. GRRs have a key driving function in the (in)stability of the SOC. We agree that testing the stability of SOC as a concept over two radically different contexts is problematic because the GRR are change. In the reviewer’s words: ‘...**participants in your study has experienced immeasurable dynamics of GRRs. In my opinion, this explains the discrepancy between pre- and post-assignment SOC in promoting health.’.** We have adapted the manuscript to ensure this point is made.

We also think it is relevant for operational reasons to highlight the change of SOC as research finding from our study. In our study we include the concept SOC as a mechanism that involves 3 processes that are involved in the longitudinal changes of the different study indicators. By showing how these three (flexible) dimensions of SOC are correlated with the changes of study variables in time we can define and explain different profiles and their changes over time.

Defining that the concept SOC is robust over time means that the 3 dimensions of SOC, e.g. Manageability, Comprehensibility and Meaningfulness are fixed processes to deal with different situations, individual resources and outcomes. By accepting that SOC is not a robust/stable construct we can explain the different response on the different ‘environmental threats, challenges and demands’. Based on our study findings we expect that future studies that specifying SOC as a flexible concept will help to better understand different longitudinal trajectories, but also can help to specify (operational) guidance, support, prevention, manage and change of deteriorated longitudinal trajectories. The change in our manuscript refers to this point also.

**Textual Change in the header Conceptualisation of SOC**

The discrepancy between pre- and post-assignment SOC in promoting health may imply that SOC is not a stable *(fixed)* construct over time as viewed by Antonovksy (3). *The instability is likely attributable to retesting in a radically different environment. The pre-assignment SOC, with its Generalised Resistance Resources such as coping strategies, emotional closeness, knowledge and intelligence, and support system (5), is not geared toward dealing with such new challenges, for example, arriving and working in a (new) humanitarian context.* *An environment that entails a different Generalised Resistance Resources (GRR), exerting their influence on SOC.*

Transposing a pre-assignment SOC to function in a novel extreme stress environment may cause health issues if SOC is not adapted to the new environment. This may explain the pre-assignment SOC health-deteriorating mechanism described earlier. It may require iHAWs time to reassess current stressors, their meaning, available resources, to create a context-appropriate SOC to deal with new challenges.

*Though SOC may be (more) stable in environments that do not change (much) over time (4). SOC, and its underlying GRR,* may be best viewed as a context-specific or situational state-like disposition rather than a trait-like disposition or global life orientation (42, 43). *By accepting that SOC is not a stable construct we can explain the different response on the different ‘environmental threats, challenges, demands, and resources (GRR). Based on our study findings we expect that future studies specify SOC as a flexible concept which will help to improve understanding of the different longitudinal trajectories. More importantly, it can help to specify guidance on supportive interventions, prevention measures, individual case management of those with deteriorated longitudinal trajectories*.
